# Dynamic responses of gross primary productivity to compound hot extremes and drought across different geographical regions of China

**DOI:** 10.3389/fpls.2025.1715432

**Published:** 2026-01-12

**Authors:** Yu Qin, Qiuxiang Jiang, Youzhu Zhao, Zilong Wang, Meiyun Tao, Baohan Li

**Affiliations:** 1 School of Water Conservancy and Civil Engineering, Northeast Agricultural University, Harbin, China; 2 International Cooperation Joint Laboratory of Health in Cold Region Black Soil Habitat of the Ministry of Education, Harbin, China

**Keywords:** compound hot extremes and drought, GPP, STI, SPEI, geographical detector

## Abstract

**Introduction:**

Amid the escalating challenges of global climate change, the stress effects of compound extreme climate events on terrestrial ecosystems are becoming increasingly prominent. However, a critical knowledge gap persists in quantitatively dissecting their synergistic impact on vegetation gross primary productivity (GPP).

**Methods:**

This study introduces a novel framework that integrates spatiotemporal trend analysis, correlation methods, and the geographical detector model. This integrated approach is designed to quantify the non-linear interactions between compound hot extremes and drought events, and GPP across China, and to systematically reveal the interaction mechanisms among their characterizing indicators—namely, the Standardized Temperature Index (STI), the Standardized Precipitation Evapotranspiration Index (SPEI), and GPP.

**Results:**

The results show that: (1) From 2001 to 2020, the interannual variation of GPP in China's six major regions showed an increasing trend, 98.84% have experienced significant warming, and 83.76% show no significant changes in drought. (2) The occurrence of compound hot extremes and drought events shows significant regional heterogeneity, with a frequency of 14.4% in North China and 13.1% in Northwest China. Compound hot extremes and drought events exhibit distinct regional heterogeneity, being most frequent in North China and least frequent in Northwest China. (3) Correlation analysis indicates that 72.4% of regions show a negative correlation between STI and GPP; 64.1% show a positive correlation between SPEI and GPP; and 71.69% show a negative correlation between the frequency of compound hot extremes and drought events and GPP. (4) Interaction effect analysis highlights that the impact of compound hot extremes and drought events on GPP exceeds that of either factor alone, with the most significant effect in North China (q values of STI, SPEI, and compound hot extremes and drought events are 0.14, 0.25, and 0.51, respectively. Moreover, the interaction exhibits a synergistic amplification.

**Discussion:**

This study provides new data support for assessing ecosystem resilience and informing adaptive management under climate change.

## 1 Introduction

Gross primary productivity (GPP), a fundamental indicator of vegetation’s photosynthetic carbon uptake, plays a central role in regulating ecosystem carbon cycling (Cao et al., 2022; Qiu et al., 2020; Zhang and Ye, 2022). As a primary driver of ecosystem functions, the spatiotemporal dynamic changes of GPP directly affect the regional and even global carbon budget balance (Anav et al., 2015). In recent years, the increasing frequency of extreme weather events, GPP has shown significant fluctuation characteristics. Severe high temperatures and droughts usually have a certain degree of negative impact on GPP (Li et al., 2023; Piao et al., 2019). Studies show that between 2000 and 2011, following widespread droughts worldwide, the Northern Hemisphere’s mid-latitude regions experienced a dramatic drop in GPP—plummeting by nearly half (48%); Meanwhile, the Southern Hemisphere’s tropical zones saw a much milder decrease of just 13% (Yu et al., 2017). In addition, extreme meteorological events have had a significant impact on China. Research indicates that extreme meteorological disasters have led to a decrease of approximately 2.3% in China’s GPP. Among these events, high temperatures and droughts have had a greater impact in Southern China, accounting for 60% to 70% of the total disasters, while in Northern China, the proportion is 30% to 40% (Chen et al., 2019). This significant regional disparity highlights the important scientific value and practical significance of in-depth research on the mechanism by which high-temperature and drought stress affect GPP.

During the period from 2000 to 2020, China experienced several severe droughts, with 2001 and 2011 being typical drought years (Cao et al., 2023). Similarly, high-temperature stress is also an important factor affecting vegetation GPP. But under normal circumstances, high-temperature and drought events do not occur separately in most regions; they usually occur simultaneously or successively. When a region experiences high-temperature and drought events simultaneously or in sequence, it is referred to as a compound hot extremes and drought event (Wang et al., 2023). Such compound hot extremes and drought events have a more severe negative impact on GPP.

Under the backdrop of global climate change, the superimposed effects of frequent compound hot extremes and drought on ecosystems have become a key research topic. Wu and Jiang (2022) found that in regions with a high frequency of compound hot extremes and drought events globally, the inhibitory effect on GPP is more pronounced. Ciais et al. (2005) found that Europe’s 2003 extreme heatwaves and drought sharply decreased GPP. Comparative studies assessing the impacts of single drought conditions and compound hot extremes and drought events on ecosystems, it was confirmed that compound events result in a more pronounced downward trend in GPP, with a decline significantly greater than that caused by single drought conditions (von Buttlar et al., 2018). The synergistic interaction between heat and drought intensifies vegetation evaporation, reduces precipitation, and increases vegetation water demand. In 2022, Southern China experienced a severe compound hot extremes and drought event that lasted for a long time and was of high intensity, causing GPP to drop by 10% compared to the average level from 2001 to 2021 (Zhang et al., 2024), resulting in significant economic losses. Moreover, compound hot extremes and drought events significantly prolong the recovery time of ecosystems. Research indicates that recovery in arid regions takes 1.5 months longer than in humid regions, and globally, areas affected by compound hot extremes and drought events recover 5 months later than those impacted by single drought or heat events (Yao et al., 2024). Understanding how GPP responds to compound hot extremes and drought conditions is crucial—it offers both scientific insights and actionable strategies for effective ecosystem conservation and recovery efforts.

Existing studies have employed various statistical methods to analyze the correlations between drought and heat indices and vegetation indices. Hao et al. (2021) systematically evaluated the correlation between global standardized precipitation index (SPI) and standardized temperature index (STI) and NDVI based on Pearson correlation analysis. Li et al. (2024) used the geodetector method to systematically assess the single-factor and interaction effects on NPP in China. Therefore, this study integrates Pearson correlation analysis and the geodetector to construct a multi-scale analysis framework, systematically revealing the dynamic response mechanism of vegetation GPP in different geographical regions of China to compound heat and drought events, thereby establishing a connection between point correlation and overall impact patterns, and providing new research methods and perspectives for understanding the interaction between climate and vegetation.

This study employs the STI and the SPEI as the characterization indicators for compound hot extremes and drought events. The research framework consists of four key steps: (1) spatiotemporal pattern analysis of STI, SPEI, and GPP; (2) identification and frequency calculation of compound hot extremes and drought events based on STI and SPEI thresholds; (3) correlation analysis between hot extremes, drought, compound events, and GPP; (4) driving factors analysis of compound hot extremes and drought effects on GPP. The scientific question of this study lies in: How to systematically reveal the impact mechanism of compound hot and drought events on the GPP of vegetation at the regional scale in China, especially to quantify the independent and interactive effects of key driving factors?

## 2 Data and methods

### 2.1 Study area

**Figure 1:**
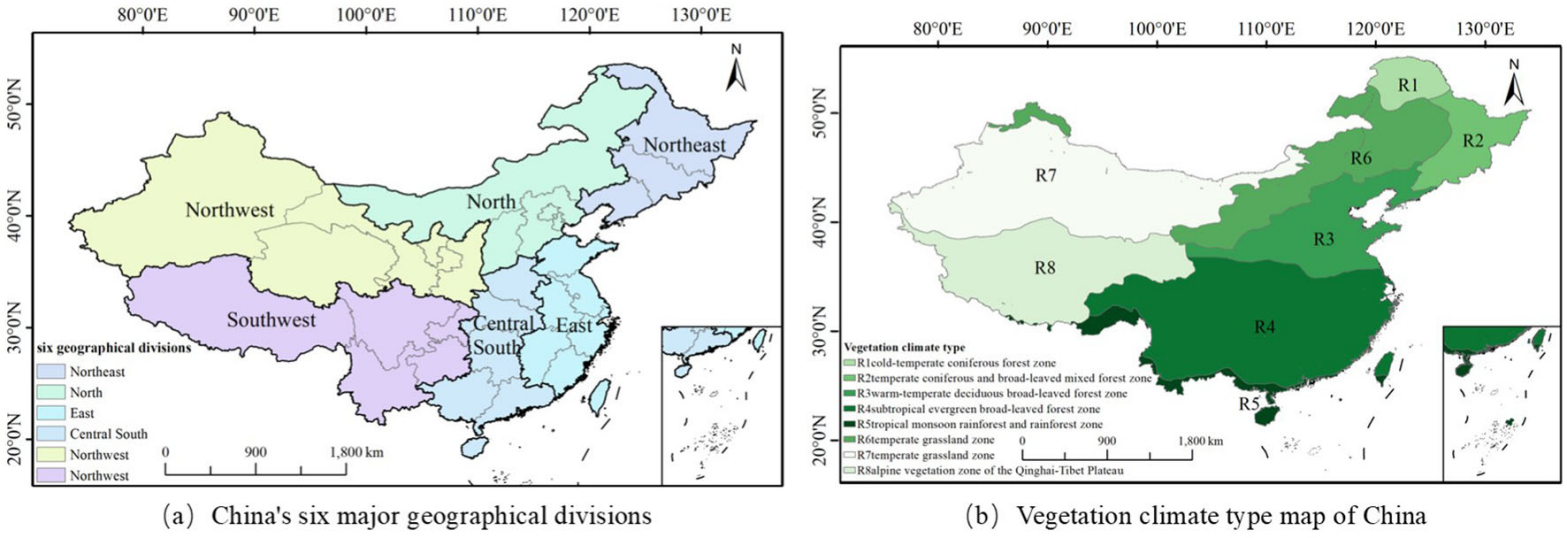
Shows the regional map of China.

Situated in the Eastern part of the Asian continent, China faces the western Pacific Ocean, with geographical coordinates ranging from 3°51′N to 53°33′N latitude and 73°33′E to 135°05′E longitude (Wu et al., 2020). The country covers approximately 9.6 million square kilometers of land area, stretching about 5,000 kilometers from east to west, and has a continental coastline exceeding 18,000 kilometers (Wang et al., 2024). Given China’s vast territory and significant geographical span, this study follows the national basic geographical division framework and defines the research area as six major geographical regions: Northeast China, North China, East China, Central and South China, Northwest China, and Southwest China (**Figure 1a**). Vegetation distribution patterns reveal that the Northeast region is primarily composed of temperate coniferous and broad-leaved mixed forests, North China is characterized by temperate grasslands, East China and Central and South China mainly feature warm temperate deciduous broad-leaved forests and subtropical evergreen broad-leaved forests, Northwest China is dominated by temperate grasslands, and Southwest China encompasses the alpine vegetation zone of the Qinghai-Tibet Plateau and subtropical evergreen broad-leaved forests (**Figure 1b**). This vegetation distribution gradient creates a spatial pattern where China’s total GPP increases progressively from the Northwest to the Southeast. Nevertheless, the increasing frequency of compound hot extremes and drought events in recent years has not only heightened GPP variability but also posed significant risks to regional ecosystem stability (von Buttlar et al., 2018). Consequently, a thorough investigation into the impact mechanisms of compound hot extremes and drought events on GPP holds substantial scientific and practical importance for comprehending ecosystem response patterns under climate change and developing adaptive management strategies.

### 2.2 Data

#### 2.2.1 MODIS GPP data

The MODIS dataset, derived from the light use efficiency model, operates on a daily scale. In this study, the GPP data provided by the MOD17A2 product of MODIS (https://lpdaac.usgs.gov/products/mod17a2hgfv061/) was used. This dataset features an 8-day temporal resolution and a spatial resolution of 0.5° (Zheng et al., 2024). Currently, MODIS GPP data is widely applied in ecosystem carbon cycling and global change research. In this study, the 8-day GPP data was synthesized into monthly-scale data, and the data for the study area was obtained through clipping and resampling.

#### 2.2.2 SPEI data

The Standardized Precipitation Evapotranspiration Index (SPEI) is one of the most commonly used indices for identifying meteorological droughts, which can represent the severity of drought or the degree of moisture in a region. SPEI is an index calculated by combining precipitation and evapotranspiration conditions and has multiple time scales. It is widely used by scholars worldwide (Luo et al., 2020). To align with the monthly GPP data, this study utilized SPEI data spanning from 2001 to 2020, with a monthly temporal resolution, obtained from SPEIbase v.2.8 dataset (https://spei.csic.es/database.html). According to the value of SPEI and existing research, this study defined drought occurrence as SPEI ≤ -0.5. This threshold corresponds to the onset of drought conditions, which, while not reaching extreme drought levels, effectively captures the early stage when water availability begins to exert stress on vegetation. Selecting this threshold aims to systematically identify events ranging from mild to extreme drought, thereby providing a more comprehensive assessment of the ecological impacts of compound events (Wu et al., 2023).

#### 2.2.3 Temperature data

The monthly average temperature data for the period 2001–2020 were obtained from the National Tibetan Plateau Data Center (https://data.tpdc.ac.cn/home) (Peng, 2019), with a spatial resolution of 0.5°.

### 2.3 Methods

#### 2.3.1 Identification of compound hot extremes and drought conditions

This study uses the combination of the SPEI and the STI to represent the situation of compound hot extremes and drought.

Since the abnormal temperature can be assumed to follow a normal distribution, the standard normal distribution can be directly fitted to each detrended monthly temperature time series, and finally the quantile function of the standard normal distribution of the monthly temperature time series is calculated (Zscheischler et al., 2014). The formulation for STI is presented as follows in Equations 1, 2:

(1) $$STI=\varphi^{-1}(P)$$

(2) $$P=\frac{1}{\sigma \sqrt{2\pi }}\int_{-\infty }^{T}\exp(-\frac{{\left(t-\mu \right)}^{2}}{2\sigma^{2}})dt$$

In the formula: *P* represents the cumulative probability; while *φ* denotes the standard normal distribution function; The variable *t* i corresponds to the temporal sequence of monthly mean temperatures; with *μ* and *σ* representing the arithmetic mean and standard deviation of this sequence, respectively.

This investigation characterizes compound hot extremes and drought as the concurrent manifestation of elevated high temperature and drought in a region. This study defines STI > 0.5 as a heat event. This threshold corresponds to the onset of ‘mild heat, indicating that the temperature has significantly deviated from the long-term average. To maintain statistical symmetry with the defined drought threshold (SPEI ≤ -0.5) and systematically identify the compound event type, which has a high occurrence frequency and ecological significance, STI > 0.5 is therefore used as the heat threshold (Wu et al., 2021). Therefore, in this study, regions with STI > 0.5 and SPEI< -0.5 are marked as experiencing compound hot extremes and drought. A month during which a compound hot extremes and drought event occurs is counted as one instance. The frequency of compound hot extremes and drought is calculated as the proportion of months that met the compound event criteria out of a total of 240 months from 2001 to 2020.

#### 2.3.2 Trend analysis

The research utilized the Theil-Sen Median technique along with the Mann-Kendall test to examine the spatial distribution patterns of STI, SPEI, and GPP spanning the years 2001 to 2020. Known alternatively as the Sen slope estimator, the Theil-Sen Median method represents a nonparametric statistical approach frequently employed in the analysis of trends within long-term time series datasets (Tian et al., 2021). This method determines the trend of data through the parameter *β*, where a positive *β* value signifies an increasing trend and a negative *β* value denotes a decreasing trend. The calculation of *β* is expressed by the following formula (Equation 3):

(3) $$\beta =\text{Median}(\frac{SPEI_{j}-SPEI_{i}}{j-i}),\text{ ∀}j>i$$

In the formula: *SPEI_i_* and *SPEI_j_* respectively denote the standardized precipitation evapotranspiration Index values for the i-th and j-th months.

The Mann-Kendall test is a non-parametric statistical test method, which is suitable for analyzing the trend of long-term series data. Its advantage lies in the fact that it does not require the data to follow a normal distribution and is applicable to trend tests with missing or abnormal data (Cui et al., 2023). The Mann-Kendall test uses the test statistic Z for trend testing, and the formula for calculating the Z value is as follows in Equations 4-7:

(4) $$Z=\left\{ \begin{array}{c} \frac{S-1}{\sqrt{Var(S)}},\;(S>0)\\ \frac{S+1}{\sqrt{Var(S)}},\;(S<0) \end{array}\right.$$

(5) $$S=\sum_{i=1}^{n-1}\sum_{j=i+1}^{n}\mathrm{sgn}(x_{j}-x_{i})$$

(6) $$\mathrm{sgn}(x_{j}-x_{i})=\{\begin{array}{c} 1,(x_{j}-x_{i})>0\\ 0,(x_{j}-x_{i})=0\\ -1,(x_{j}-x_{i})<0 \end{array}$$

(7) $$V\text{ar}(S)=\frac{n(n-1)(2n+5)}{18}$$

In the formula: *Z* is the standardized SPEI test statistic. When |*Z*| ≥ 1.96, the dataset indicates a notable trend; when |*Z*|< 1.96, the trend is considered insignificant; *S* is the SPEI test statistic of vegetation; sgn is the sign function; *Var*(*S*) signifies the variance; *n* represents the temporal scal, which in this research is set at 24.

#### 2.3.3 Pearson correlation analysis

In contemporary research focusing on relationship assessment, Pearson’s correlation methodology stands out as a predominant analytical approach. This statistical measure efficiently quantifies both the strength and orientation of associations, thereby illuminating the interconnections among diverse components (Fan et al., 2024). Within the framework of this investigation, Pearson’s analytical technique was employed to examine the interrelations among monthly STI, SPEI, and GPP. The mathematical expression for determining the correlation coefficient rxy in Pearson’s analysis is presented below (Equations 8, 9):

(8) $$r_{xy}=\frac{\sum_{i=1}^{n}\left(x_{i}-\bar{x})(y_{i}-\bar{y}\right)}{\sqrt{\sum_{i=1}^{n}{\left(x_{i}-\bar{x}\right)}^{2}}\sqrt{\sum_{i=1}^{n}{\left(y_{i}-\bar{y}\right)}^{2}}}$$

(9) $$r_{xy-z}=\frac{r_{xy}-r_{xz}r_{yz}}{\sqrt{\left(1-r_{xz}^{2})(1-r_{yz}^{2}\right)}}$$

In the formula: *r_xy_* represents the correlation coefficient between elements *x* and *y*. A positive value indicates a positive correlation, a negative value indicates a negative correlation, with the magnitude reflecting the intensity of this connection; *x_i_* and *y_i_* denote the respective measurements of *x* and *y* during the i-th month; $\bar{x}\;$ and *ȳ* stands for the mean values of both variables across the temporal sequence; *n* ndicates the total count of years or months under consideration; *r_xy_*_-_*_z_* represents the partial correlation coefficient between the initial two variables when the third is held constant; *r_xy_*, *r_xz_*, and *r_yz_* correspond to the pairwise correlation coefficients among the trio of variables *x*, *y*, and *z*.

#### 2.3.4 Geographical detector

The Geodetector serves as a statistical tool designed to examine the spatial variability of geographical phenomena and identify their underlying drivers (Wang and Xu, 2017). This method evaluates the spatial distribution patterns of geographical elements, uncovering the extent to which various factors impact these phenomena and how they interact (Zhang et al., 2021). Traditional geographical detector models often rely on the discretization of continuous variables during operation, and the discretization parameters are mostly subjectively determined by researchers, which can easily lead to unstable results. To address this, this study adopts the improved optimal parameter-based geographical detector model proposed by Song et al (2020). This model automatically determines the optimal discretization parameters that maximize the explanatory power (q-statistic) of factors, thereby effectively enhancing the objectivity and robustness of the results (Li et al., 2025). Based on this, the study primarily applies its factor detection and interaction detection functions to conduct the analysis.

The single-factor detector evaluates the effect of an independent variable *X* on a dependent variable *Y*, quantified through the q value (Wang and Xu, 2017). A larger *q* value signifies a more substantial influence of *X* on *Y*, whereas a smaller *q* value denotes a lesser impact. The formula used to compute the *q* value is Equation 10:

(10) $$q=1-\frac{1}{N\sigma^{2}}\sum_{h=1}^{L}N_{h}\sigma_{h}^{2}$$

In the formula: *N* denotes the complete sample size of the population; *σ*^2^ signifies the overall variance of vegetation GPP; indicates the stratification of natural factor *X* or vegetation GPP; *L* represents the total number of stratifications for natural factor *X* or vegetation GPP; *N_h_* stands for the sample size of the *h*th stratum; *σ*2 *h* denotes the variance of the natural factor within the *h*th stratum.

Interaction detection can identify the extent to which the combined effects of different factors influence GPP. That is, by comparing the magnitudes of *q*(*X*_1_),*q*(*X*_2_), and *q*(*X*_1_∩*X*_2_), it can analyze the strength of the individual and combined effects of factors on GPP. The criteria for judging interaction effects are shown in **Table 1**.

**Table 1:** Types of Interaction Detection.

Criterion for discrimination	Type of interaction	Number
*q*(*X*_1_∩*X*_2_)<Min[*q*(*X*_1_), *q*(*X*_2_)]	Nonlinear Weakening	I
Min[*q*(*X*_1_), *q*(*X*_2_)]<*q*(*X*_1_∩*X*_2_)<Max[*q*(*X*_1_), *q*(*X*_2_)]	Single-Factor Nonlinear Weakening	II
*q*(*X*_1_∩*X*_2_)>Max[*q*(*X*_1_), *q*(*X*_2_)]	Two-Factor Enhancement	III
*q*(*X*_1_∩*X*_2_)=*q*(*X*_1_)+*q*(*X*_2_)	Independent	IV
*q*(*X*_1_∩*X*_2_)>*q*(*X*_1_)+*q*(*X*_2_)	Nonlinear Enhancement	V

## 3 Result

### 3.1 The spatiotemporal variation characteristics of GPP, high temperature and drought

**Figure 2:**
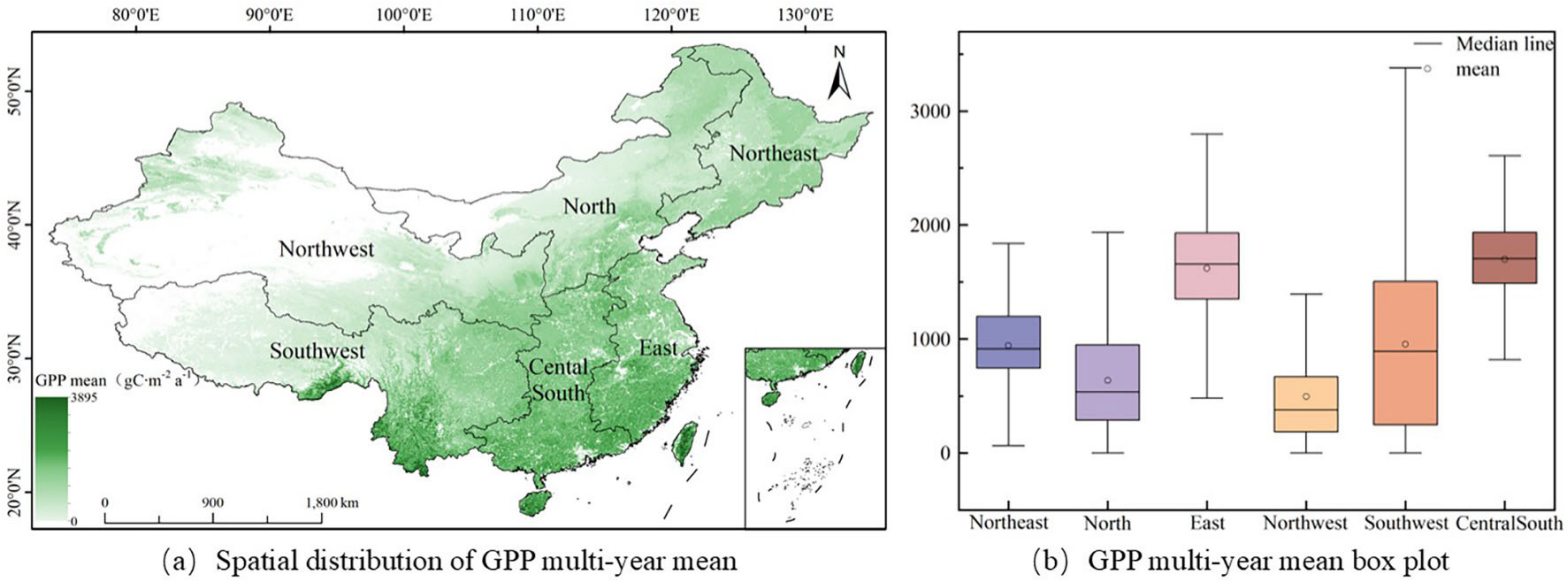
Spatial distribution of the mean GPP in China from 2001 to 2020 and statistical characteristics of GPP in each geographical region.

**Figure 3:**
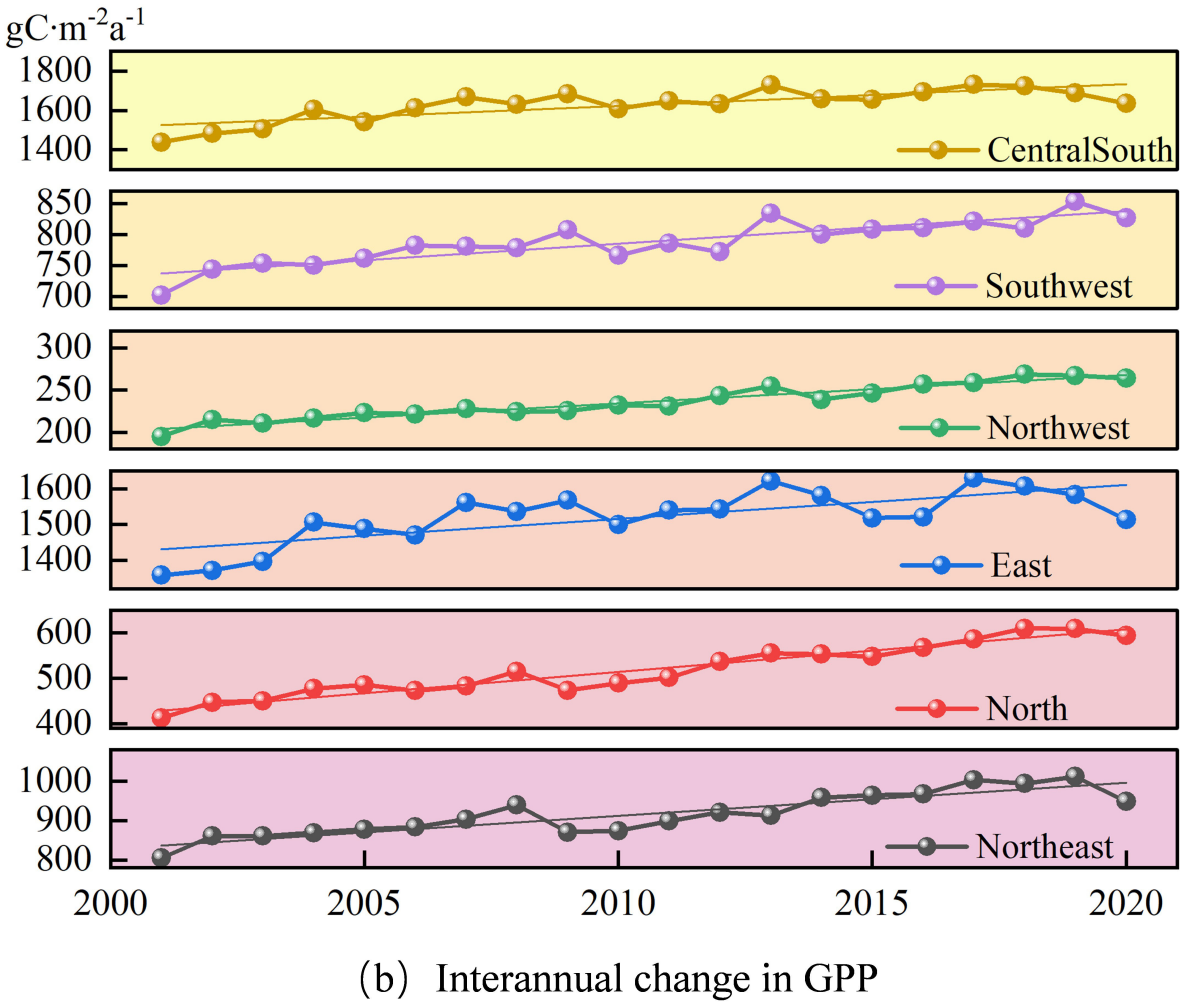
Temporal variation characteristics of GPP in different geographical regions of China from 2001 to 2020.

**Figure 4:**
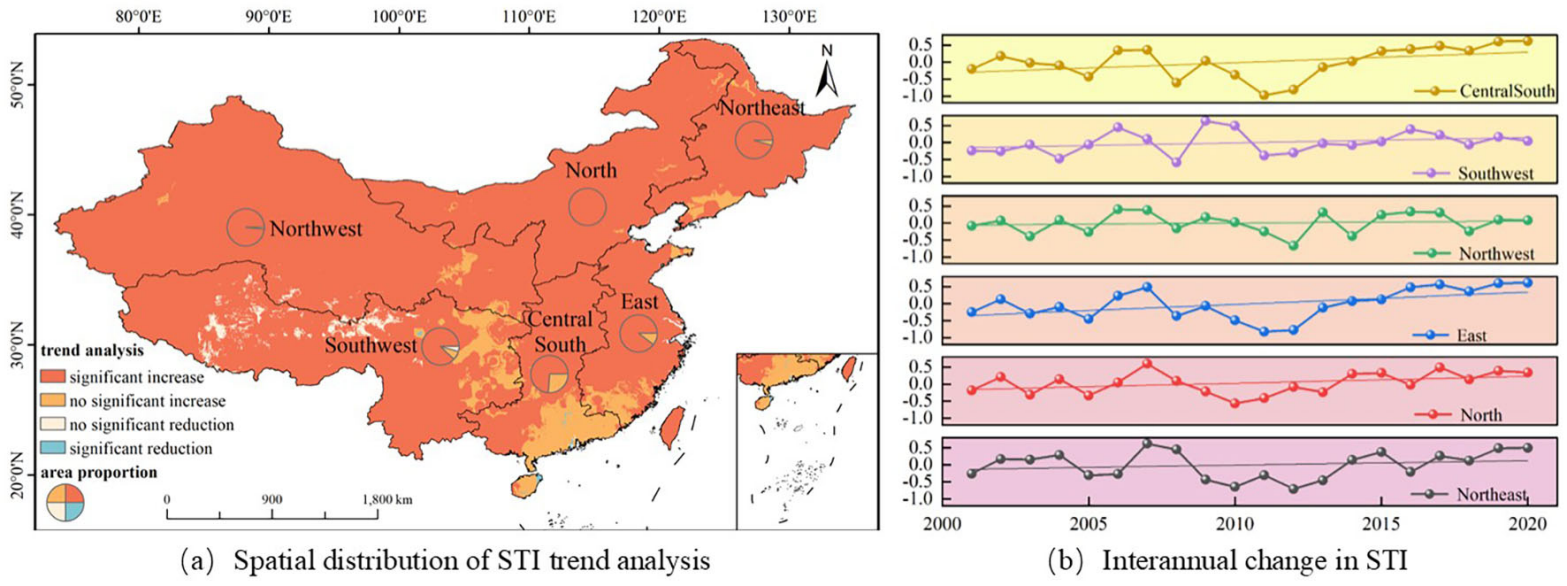
Spatial and temporal variation characteristics of STI in various geographical regions of China from 2001 to 2020.

This paper uses the Theil-Sen Median method and the Mann-Kendall method to analyze the spatial variation trends of GPP, STI and SPEI from 2001 to 2020, calculates the interannual variations of GPP, STI and SPEI from 2001 to 2020, and plots their spatial and interannual variation maps (see **Figures 2**–**4**).

#### 3.1.1 The spatiotemporal variation characteristics of GPP

From 2001 to 2020, the mean GPP of vegetation in China was 1057.5 gC·m^-^²a^-1^. Spatially, GPP exhibited a distinct gradient, with lower values observed in the Northwest and higher values in the Southeast (**Figure 2a**). Notably, the Central South and East China regions displayed significantly elevated GPP levels compared to other areas, while the Northwest region had the lowest GPP. The ranking of mean GPP in the six major geographical regions was as follows: Central South (1698.3 gC·m^-^²a^-1^) > East China (1620.6 gC·m^-^²a^-1^) > Southwest (954.3 gC·m^-^²a^-1^) > Northeast (941.7 gC·m^-^²a^-1^) > North China (635.7 gC·m^-^²a^-1^) > Northwest (494.2 gC·m^-^²a^-1^). This spatial distribution highlights the heterogeneity in vegetation productivity across China.

Further analysis was conducted on the distribution range of the upper quartile (Q1) and lower quartile (Q3) of GPP in each region. As shown in **Figure 2b**, the GPP values in the Northeast region ranged from 744.7 gC·m^-^²a^-1^ to 1198.5 gC·m^-^²a^-1^, in the North China region from 288.5 gC·m^-^²a^-1^ to 947.4 gC·m^-^²a^-1^, in the East China region from 1349.5 gC·m^-^²a^-1^ to 1929.6 gC·m^-^²a^-1^, in the Northwest region from 186.0 gC·m^-^²a^-1^ to 688.7 gC·m^-^²a^-1^, in the Southwest region from 246.5 gC·m^-^²a^-1^ to 1504.8 gC·m^-^²a^-1^, and in the Central South region from 1487.8 gC·m^-^²a^-1^ to 1935.2 gC·m^-^²a^-1^. Notably, GPP in the Southwest region showed significant variation, with a considerable difference between Q1 and Q3. This is mainly attributed to the complex and diverse vegetation types in this region. The Southwest region encompasses two typical ecosystems: the alpine vegetation zone of the Qinghai-Tibet Plateau and the subtropical evergreen broad-leaved forest. Due to its high altitude, low vegetation coverage and grass-dominated vegetation structure, the GPP value in the Qinghai-Tibet Plateau is generally low, at 249.9 gC·m^-^²a^-1^; while in the subtropical evergreen broad-leaved forest region, with its dense vegetation and vigorous photosynthesis, the GPP value is significantly higher, at 1267.7 gC·m^-^²a^-1^.This spatial heterogeneity in vegetation types directly contributes to the wide variation in GPP observe in the Southwest region.

**Figure 3** reveals that the annual GPP variation across China’s six primary geographical regions trended upwards. Among them, the Central and South region had the highest growth rate, reaching 9.88 gC·m^-^²a^-1^ per year; the North China region followed closely with a growth rate of 9.07 gC·m^-^²a^-1^ per year; the Northwest region had the lowest growth rate, only 3.46 gC·m^-^²a^-1^ per year. The growth rates in other regions were all above 6.20 gC·m^-^²a^-1^ per year. In conclusion, the GPP of vegetation in China as a whole has shown a year-on-year increasing trend.

#### 3.1.2 The spatiotemporal variation characteristics of STI

Amid the escalating global climate crisis, China’s STI between 2001 and 2020 exhibited distinct spatial response patterns, as illustrated in **Figure 4a**. The research results show that 90.77% of the regions in China have a significantly increasing trend in STI; 6.34% of the regions have a non-significantly increasing trend; 1.88% of the regions have a non-significantly decreasing trend; and 1.01% of the regions have a significantly decreasing trend. This spatial distribution pattern confirmed that the land surface experienced a general warming under the background of climate warming, but local geographical factors still regulated the temperature change trend. In terms of trend type distribution, significantly warming areas dominated, while the combined proportion of insignificant changes (including increase and decrease) and significantly decreasing areas was less than 8%. This statistical characteristic indicated that the surface temperature change in China had significant climate forcing response characteristics, and local anomalies did not alter the overall warming trend.

**Figure 5:**
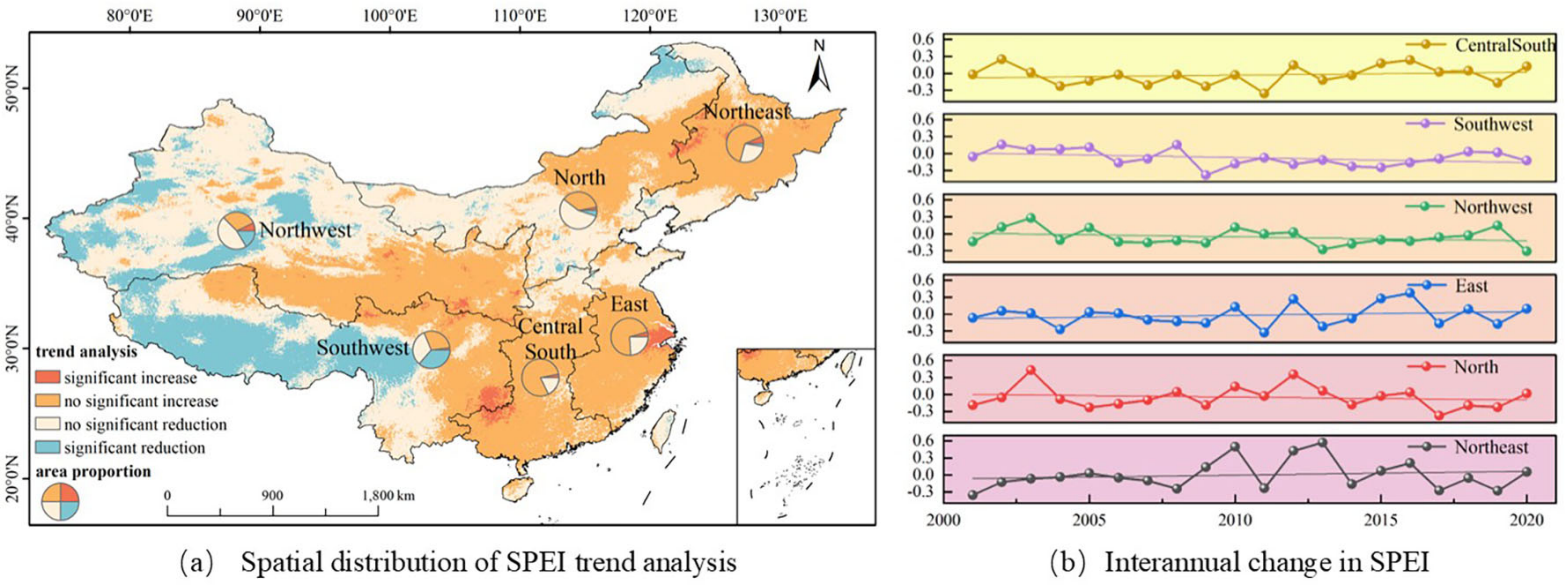
Spatiotemporal variation characteristics of SPEI in different geographical regions of China from 2001 to 2020.

An analysis of annual STI variation rates across China’s six primary geographical regions reveals consistent positive trends with notable spatial disparities, as depicted in **Figure 5b**. Among them, the Eastern China (0.043/year) and Central-Southern China (0.042/year) have the highest warming rates, significantly exceeding the national average (0.032/year); followed by the Northeastern China (0.038/year) and Northern China (0.026/year); the Southwestern China (0.014/year) and Northwestern China (0.008/year) have relatively lower warming rates, indicating that China as a whole has entered a systematic warming stage.

#### 3.1.3 The spatiotemporal variation characteristics of SPEI

Based on the spatiotemporal evolution analysis of SPEI from 2001 to 2020, drought trends in China shows significant spatial heterogeneity (see **Figure 5a**). The distribution of trend patterns demonstrates that regions experiencing marked intensification (1.62%) or reduction (14.62%) in drought conditions collectively represent less than one-fifth of the total area. In contrast, areas with stable conditions,including42.92% with a minimal increase and 40.84% with a slight decrease, dominate the pattern, representing 83.76% of the territory. This statistical result indicates that despite local variations, the overall trend of drought severity in China’s terrestrial ecosystems did not increase or decrease during the period from 2001 to 2020. Geographically, the Northeast Plain along with sections of East and South China exhibited subtle upward trends, while the Northern Northwest region displayed a mild downward trend, covering 52.74% of the spatial distribution. However, the Southwest region showed a significant decrease trend, with a spatial distribution proportion of 36.42%, suggesting that this region may be more significantly affected by drought.

The interannual variation analysis of SPEI across China six major geographical regions further supports this conclusion (see **Figure 5b**). The annual change rates of SPEI in each region remain within the range of ±0.02/year, with the Northeast region having the largest change rate (0.020/year), followed by North China (0.010/year) and East China (0.008/year). Although the SPEI in Northwest (-0.008/year) and Southwest regions (-0.003/year) show a negative trend, the magnitude of the change is very small. The interannual variation trend lines of SPEI in each region further indicate that the severity of drought severity in China has remained relatively stable throughout the study period, with annual SPEI fluctuations maintaining a consistently low amplitude.

### 3.2 The frequency of compound hot extremes and drought

**Figure 6:**
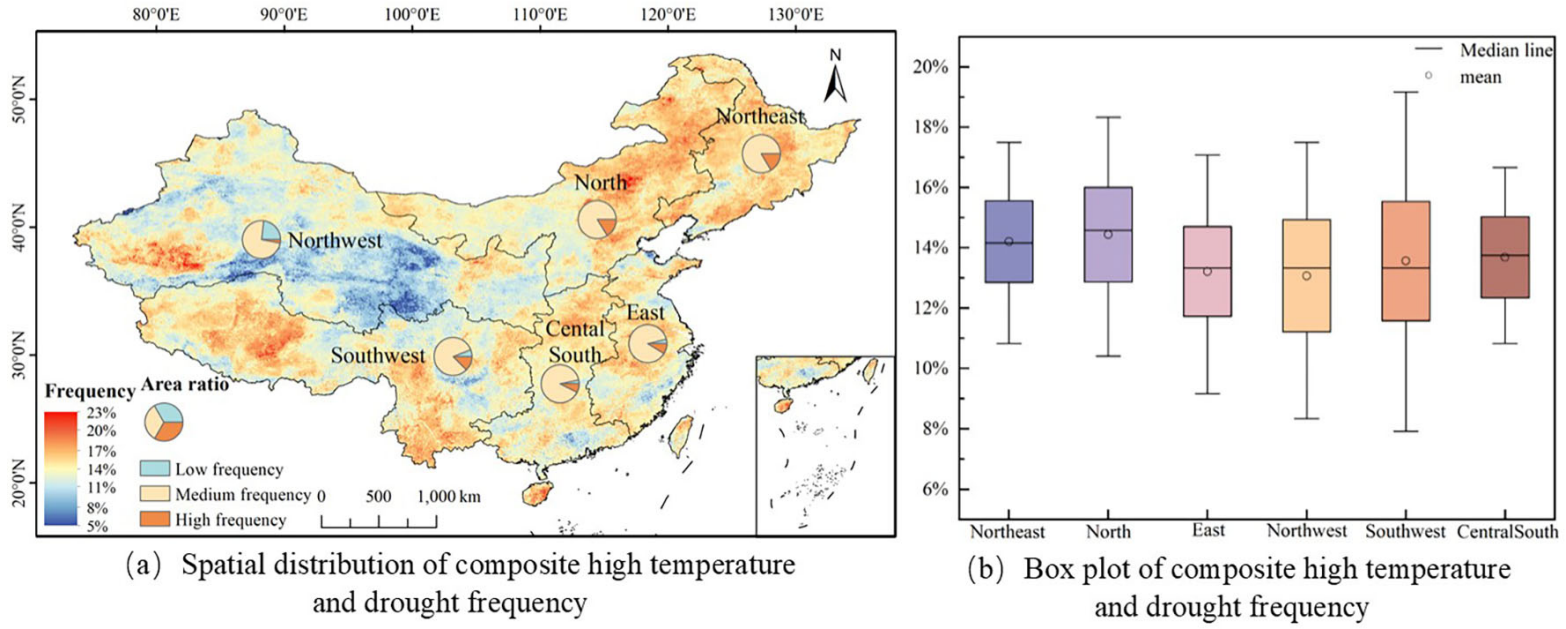
Frequency of compound hot extremes and drought in China from 2001 to 2020.

To evaluate the specific conditions of compound hot extremes and drought, the frequency of such events in China’s six major geographical regions from 2001 to 2020 was calculated based on the criteria STI > 0.5 and SPEI< -0.5 (see **Figure 6**).

The analysis of compound hot extremes and drought occurrences across China from 2001 to 2020 reveals a frequency spectrum ranging from 5% to 23%. The frequency is divided into three grades: low (5%-11%), medium (11%-17%), and high (17%-23%). As shown in **Figure 6a**, the proportion of medium-grade areas in Northeast, North China, East China, Northwest, Southwest, and Central South regions is relatively high, reaching 82.8%, 83.2%, 87.6%, 73.6%, 81.5%, and 89.4% respectively. The proportion of low and high grades is relatively low. The proportion of high-grade areas in Northeast China is relatively low (16.4%), indicating that the overall impact of compound hot extremes and drought in Northeast China is relatively small. In Northwest China, the proportion of low-grade areas is the highest (23.2%). Although t compound hot extremes and drought events in Southwest China predominantly fall within the medium frequency range, the proportion of high-frequency areas remains notable at 12.5%, especially in the Qinghai-Tibet Plateau and Yunnan Province, where event frequencies reach 20% to 23%.

According to **Figure 6b**, notable differences in the occurrence frequency of compound hot extremes and droughts can be observed among China’s six major geographical regions. Specifically, North China has the highest, average frequency at 14.4%, followed by Northeast China at 14.2%. The frequencies in Central South, Southwest, East China, and Northwest China decrease successively, being 13.7%, 13.6%, 13.2%, and 13.1% respectively. Additionally, by analyzing the upper and lower quartiles of each region, a deeper understanding of the distribution range of the occurrence frequency of compound hot extremes and droughts and their regional differences can be achieved. The frequency range in Northeast China is 12.9% - 15.6%, in North China 12.9% - 16.0%, in East China 11.7% - 14.7%, in Northwest China 11.2% - 14.9%, in Southwest China 11.6% - 15.5%, and in Central South China 12.3% - 15.0%. It can be seen from this that the distribution range of the occurrence frequency of compound hot extremes and droughts in Northwest and Southwest China is relatively wide, indicating that there may be significant differences in rainfall and high-temperature drought risks at different locations within these regions.

### 3.3 The impacts of high temperature, drought and frequency on GPP

**Figure 7:**
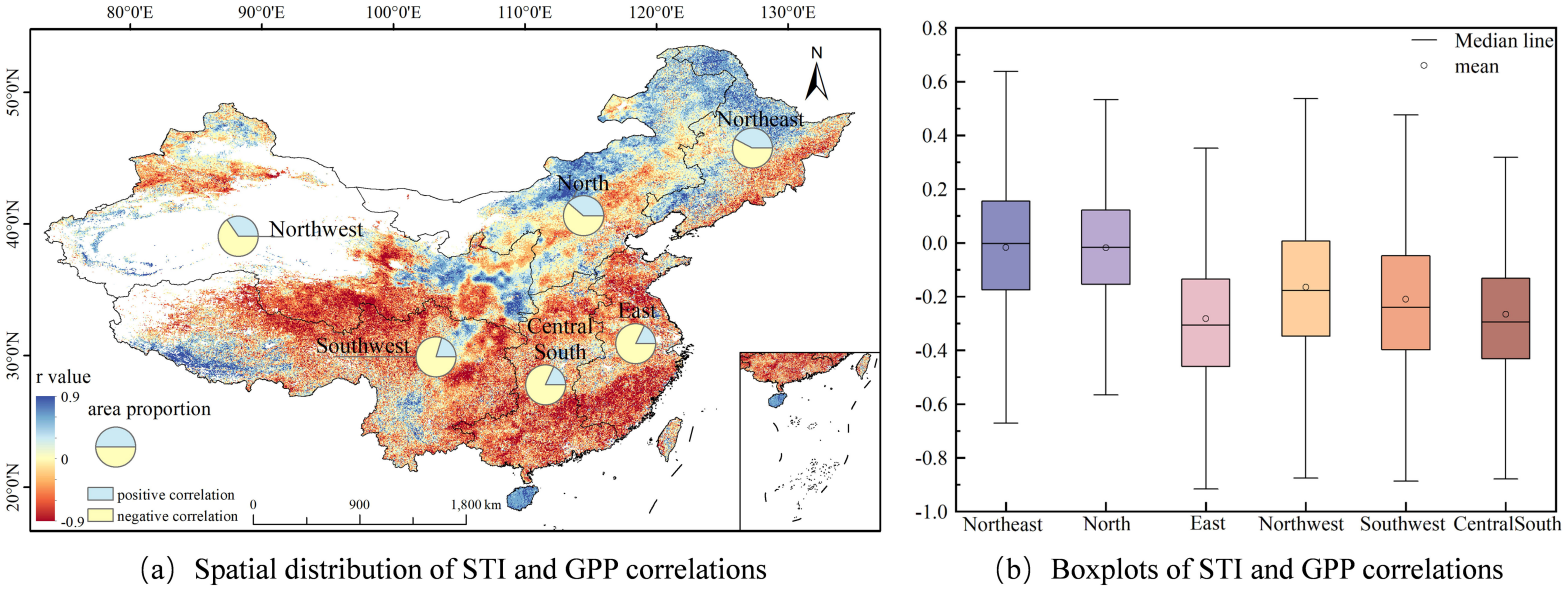
Correlation between STI and GPP.

**Figure 8:**
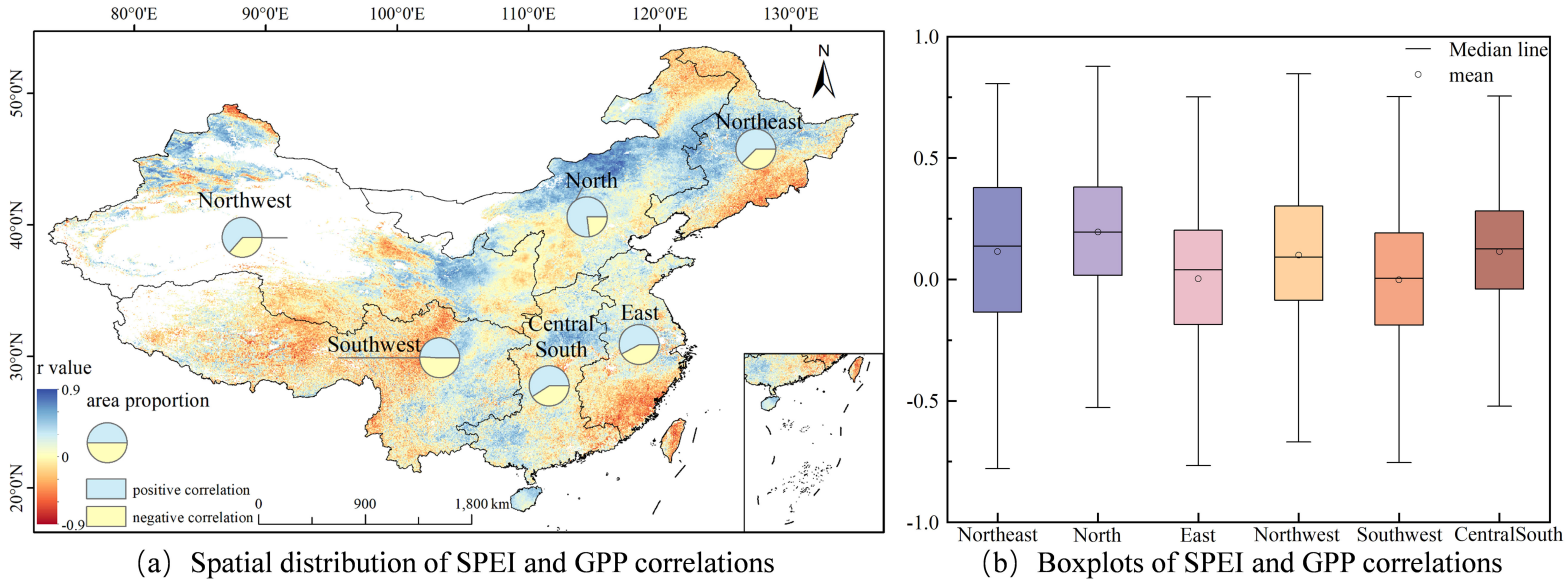
Correlation between SPEI and GPP.

This study analyzed the correlations between STI and GPP, as well as between SPEI and GPP (see **Figures 7**, **8**). The STI in China shows a significant heat stress response pattern with GPP (see **Figure 7a**). Approximately 72.4% of terrestrial ecosystems across the country show a significant negative correlation, mainly distributed in East China, Central South, and Southwest regions, with negative correlation proportions of 82.3%, 81.8%, and 79.7% respectively. This spatial distribution feature indicates that the increase in temperature generally leads to a decrease in GPP, highlighting the heat stress impact on land-based ecosystems amidst global warming. **Figure 7b** reveals that East China (r = -0.28) and Central South regions (r = -0.27) exhibit the most pronounced negative correlations; Northwest (r = -0.17) and Southwest regions (r = -0.21) follow; while Northeast (r = -0.02) and North China regions (r = -0.02) have the weakest. From each division, in the areas where STI and GPP show a negative correlation, the increase in temperature has a negative impact on GPP. The upper and lower quartiles of each region are as follows: Northeast -0.18 and 0.16, North China -0.15 and 0.12, East China -0.46 and -0.14, Northwest -0.35 and 0.01, Southwest -0.40 and -0.05, Central South -0.43 and -0.13. The significant differences in the upper and lower quartiles among regions indicate that there is considerable spatial variability in the relationship between STI and GPP across China.

As illustrated in **Figure 8a, a** substantial portion of China’s territories demonstrates a positive linkage between SPEI and GPP, with the positively correlated areas accounting for 64.1% of the country. Specifically, the Northeastern, Northern, and Northwestern regions display notably high positive correlation percentages of 62.6%, 77.0%, and 63.5% respectively. This indicates that the GPP in these regions is more sensitive to drought. The observed spatial distribution reveals a coupling relationship between drought intensity and GPP reduction, confirming the sensitivity of ecosystems to water conditions. **Figure 8b** shows, that North China has the highest average correlation coefficient (r = 0.19), followed by Northeast and Central South China (r = 0.11), while Southwest China has the weakest correlation (r = 0.01). This gradient feature is highly coupled with regional climate types - the strongly correlated areas (North China, Northwest China) mostly belong to the temperate continental climate, where the dynamics of GPP are strictly regulated by the seasonal distribution of precipitation; the weakly correlated areas (East China, Southwest China) correspond to subtropical monsoon climate zones, where the abundant annual average precipitation reduces the sensitivity of ecosystems to drought events.

**Figure 9:**
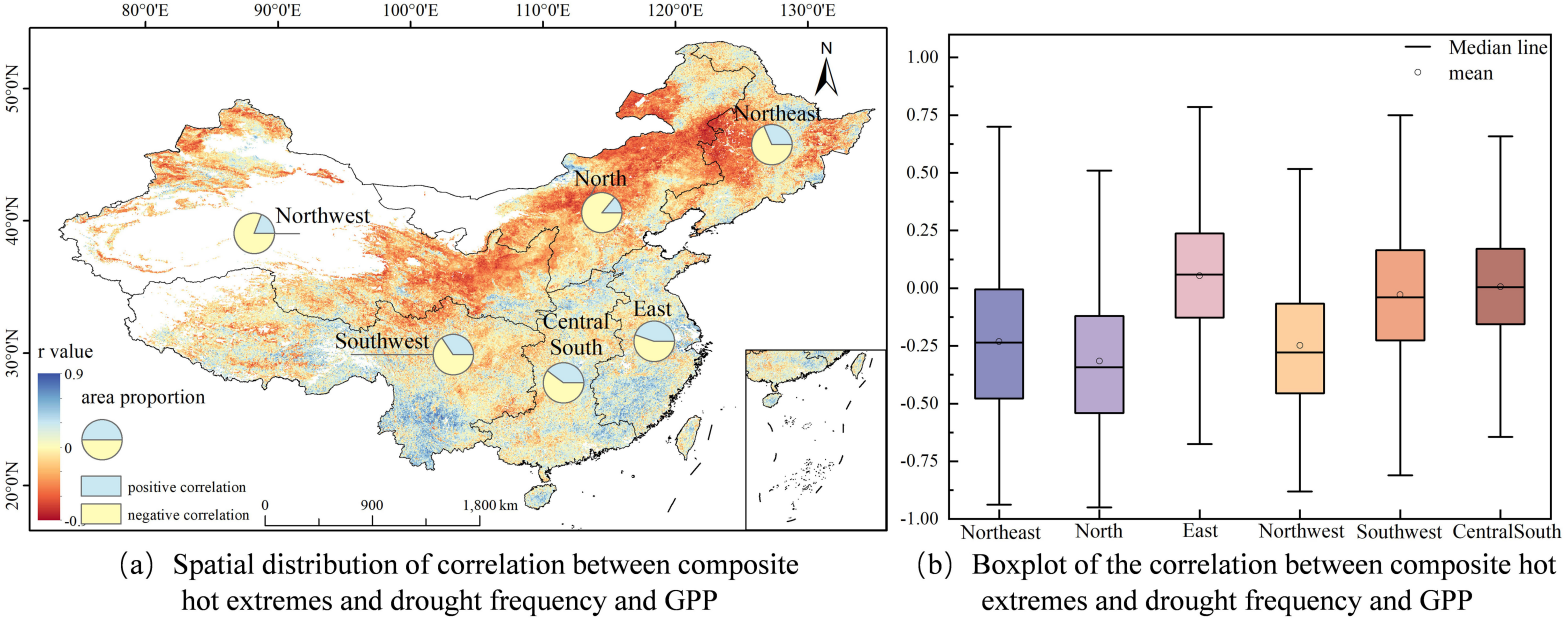
Correlation between the frequency of compound hot extremes and drought events and GPP.

**Figure 10:**
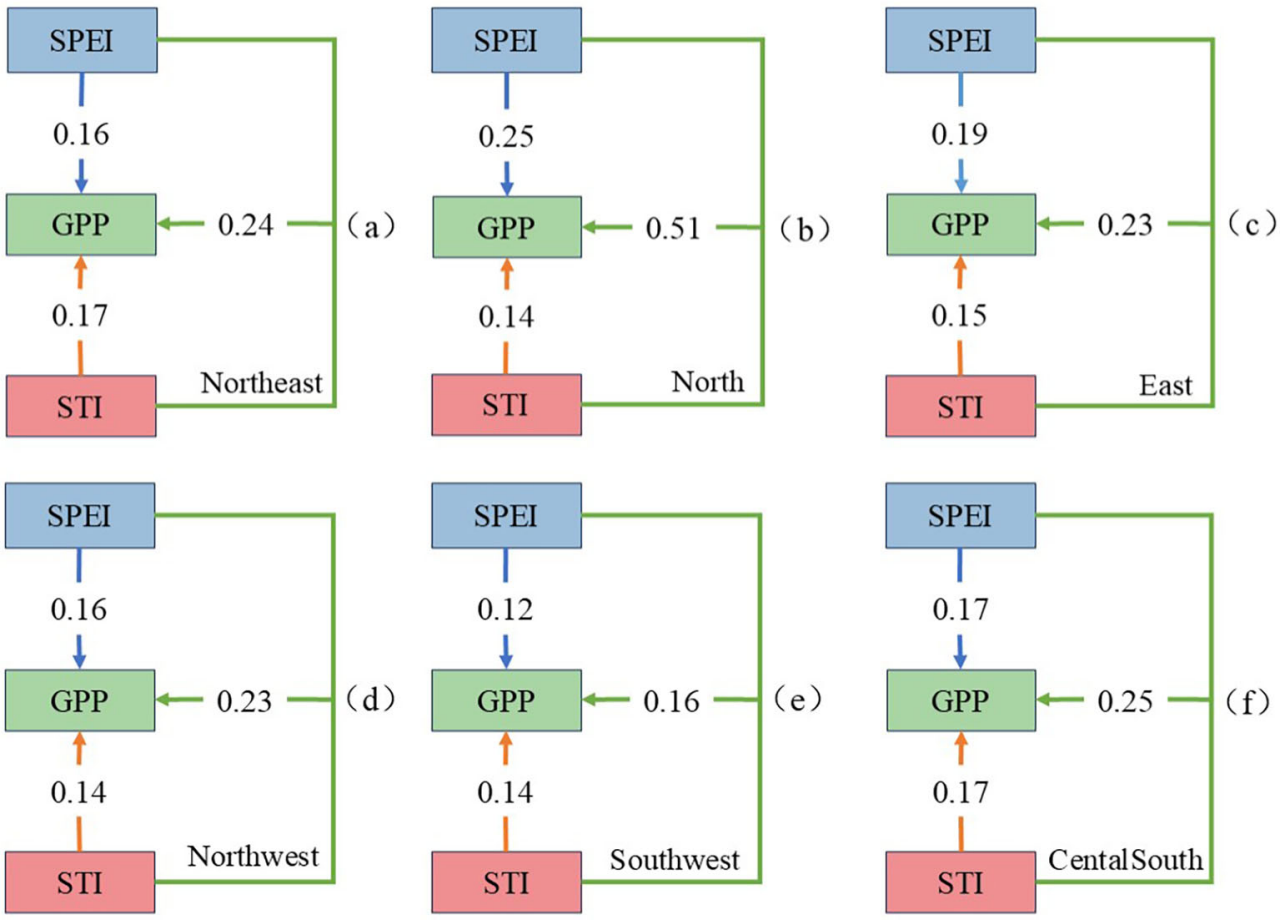
The influence degree of SPEI, STI and their interaction (compound hot extremes and drought) on GPP.

To investigate the relationship between the frequency of compound hot extremes and drought occurrences and GPP, an in-depth correlation analysis was performed to elucidate the mechanisms through which extreme climate events influence terrestrial ecosystem productivity (refer to **Figure 9**). Spatial distribution analysis revealed that 71.69% of China’s land area exhibited a pronounced negative correlation, with North China displaying an exceptionally high negative correlation rate of 86.1% (see **Figure 9a**). While Central South China exhibited lower proportions of negatively correlated areas, these still surpassed 60%, underscoring the widespread detrimental impact of compound hot extremes and drought on vegetation productivity. Further examination of the correlation strength between the frequency of compound hot extremes and drought events and GPP at a regional level (see **Figure 10b**) indicated: North China showed an extremely negative response (r = -0.32), while East China (r = 0.05) and Central and South China (r = 0.01) exhibited weak positive correlations; North China demonstrated the strongest negative correlation (r = -0.32), followed by Northeast China (r = -0.23) and Northwest China (r = -0.25). This spatial heterogeneity is related to regional ecological background conditions, with the vegetation system in these regions being more sensitive to the coupled stress of high temperature and drought.

### 3.4 The degree of influence of different factors on GPP

Utilizing the Geographical Detector’s single-factor and interaction detection methods, this study systematically analyzed the influence of drought (SPEI), high temperature (STI), and compound hot extremes and drought (SPEI∩STI) on the GPP,with results significant at the P< 0.05 level (see **Figure 10**).

The figure illustrates that North China exhibits a nonlinear enhancement interaction type, while in other regions it is double-factor enhancement. Among them, the q value of SPEI∩STI in North China is the highest (0.51), showing the strongest coupling stress effect, which is directly related to the frequent occurrence of compound hot extremes and drought events in this region from 2001 to 2020. In contrast, the q values in Northeast China, East China, Northwest China and Central South China range from 0.2 to 0.25, while the q value in Southwest China is the lowest, only 0.16. This result reveals that the impact of compound hot extremes and drought on GPP has significant regional differences. Moreover, the q values of compound hot extremes and drought events across all regions significantly surpass those of single drought (SPEI) or high temperature (STI) events, underscoring the amplified effect of the coupling stress of high temperature and drought on GPP. A deeper study of the individual impacts of drought and high temperature on GPP reveals that in Northeast China, Southwest China, and Central South China, the q value between STI and GPP exceeds that between SPEI and GPP, suggesting a more substantial influence of STI on GPP. Conversely, in North China, East China, and Northwest China, the q value between SPEI and GPP is higher than that between STI and GPP, indicating that drought (SPEI) exerts a greater impact on GPP than high temperature (STI).

## 4 Discussion

### 4.1 Trends of high temperature, drought and GPP changes

From 2001 to 2020, the examination of spatiotemporal variations in STI, SPEI, and GPP revealed distinct patterns. While STI showed a more significant increasing trend. This trend aligns with the growing incidence of extreme weather phenomena amidst global climate shifts (Liao et al., 2023). Applying the thresholds of STI > 0.5 and SPEI< -0.5, the occurrence of compound hot extremes and drought conditions across China’s six primary geographic regions was assessed. Findings show that the incidence of these compound hot extremes and drought varied between 0.05 and 0.23, with moderate occurrences (11%-17%) prevailing in most areas (84.1%-96.1%), suggesting that most areas in China are facing moderate intensity compound hot drought stress. However, regional differences remain evident, mainly reflected in the distribution pattern of high frequency events (17% - 23%). The Southwest region displays a distinct spatial pattern, with a higher prevalence of such events in the Qinghai-Tibet Plateau and Yunnan (5.5%), which is related to the thermal effect of the plateau and the variability of monsoon precipitation. In addition, the frequency distribution range in the Northwest and Southwest regions is the widest (with the largest quantile span), reflecting the high heterogeneity of internal climate-terrain factors.

Against the backdrop of increasing high-temperature and drought events, China’s vegetation GPP has shown a significant interannual growth trend. This paradoxical phenomenon reveals the complexity of ecosystem responses to climate change. Changes in GPP are regulated by the coordinated effects of multiple factors. Research by Zhang et al. (2024), focusing on temperature control, identified a strong positive link between low-level cloud cover and vegetation development. In addition, environmental factors such as relative humidity, precipitation, soil moisture, air temperature, surface temperature, and solar radiation are all important driving forces affecting vegetation GPP (Xu et al., 2024). These elements interact in complex ways, mitigating the adverse effects of drought and elevated temperatures on plant productivity. Specifically, at the regional scale, the Southeast region benefits from a warm and humid climate and rich vegetation coverage, with generally high GPP values; while the Northwest, constrained by dry and less rainy climate conditions and sparse vegetation distribution, has relatively low GPP values (Li et al., 2016). Topography further modulates the spatial distribution of vegetation productivity by influencing temperature, precipitation, and soil conditions (Xu and Zhang, 2024). This multi-scale and multi-factor combined action mechanism provides a new scientific perspective for in-depth understanding of the dynamic response patterns of vegetation under the background of climate change.

### 4.2 The impact of compound hot extremes and drought on GPP

This study analyzed the correlations between single high-temperature events, single drought events, and compound high-temperature and drought events with GPP. It was found that both drought and high temperature had negative impacts on GPP. Moreover, the geographical detector analysis highlighted that compound hot extremes and drought conditions exert a more substantial influence on GPP. This outcome implies that the effects of such compound events on vegetation GPP are more detrimental than those of singular events, aligning with the findings of Ciais et al. (2005) and von Buttlar et al. (2018). From the perspective of plant physiological mechanisms, although moderate temperatures can promote enzyme activity, when the temperature is too high, enzyme activity will be inhibited. Concurrent drought conditions further exacerbate the reduction in vegetation GPP (Wu et al., 2025). In arid zones, where water scarcity is acute, vegetation GPP becomes particularly responsive to environmental variables like precipitation and temperature (Liu et al., 2024). For example, Liu et al. (2021) in their global grassland study, observed that vegetation productivity in arid regions is highly sensitive to these climatic factors. With global warming intensifying, the frequency of compound hot extremes and drought events is escalating, intensifying their impact on vegetation GPP. Therefore, in-depth research on the impact of compound events on vegetation has significant scientific significance.

Analyzing data across China’s six major geographical regions reveals significant spatial variability in GPP’s response to high temperatures and droughts. This is mainly attributed to the unique climatic background and precipitation patterns of each region, as well as varying degrees of human intervention. Although large-scale ecological protection and restoration projects may have raised the baseline level of vegetation GPP in many areas (Xie et al., 2020), the analysis results show that 71.69% of the regions in China exhibit a significant negative correlation (p< 0.05) between the frequency of compound hot extremes and drought events and GPP, with this trend being especially pronounced in arid and semi-arid areas. Among them, the proportion of negatively correlated areas in North China is the highest (86.1%), and the correlation intensity is the greatest (r = -0.32). Considering that this region is also a key implementation area for multiple ecological restoration projects, this strong negative correlation precisely indicates that the negative impact of climatic stress in some periods and regions may be powerful enough to partially offset the vegetation growth benefits brought about by human management, confirming the intrinsic sensitivity of the ecosystem in this region to water stress (Ma et al., 2021). The geographical detector analysis further confirms that the explanatory power of compound hot extremes and drought events in North China (q = 0.51) is significantly higher than that of single drought (q = 0.25) or high temperature (q = 0.14), indicating that the synergistic effect of high temperature and drought can increase the risk of vegetation decline (Hao et al., 2021b). This result is consistent with global-scale research indicating that compound climate events amplify ecological risk (Zscheischler et al., 2018).

### 4.3 Shortcomings and Prospects

Spanning diverse geographical landscapes, China is characterized by seven distinct climatic regions, each exhibiting unique vegetation responses to high temperatures and aridity (Yao et al., 2018). However, this study did not conduct an in-depth exploration of the changing trends of GPP in each climate zone. It should be noted that vegetation GPP is influenced by a variety of environmental factors, including low temperatures, nitrogen and phosphorus nutrient supply, flood events, and the increase in atmospheric vapor pressure deficit (VPD) (Dannenberg et al., 2022). The current research scope was limited to high temperatures and drought, excluding other critical environmental stressors affecting vegetation productivity. Moreover, anthropogenic influences on vegetation GPP demand particular attention. Moreover, anthropogenic influences on vegetation GPP demand particular attention (Liu et al., 2024). Conversely, the Northwestern and Qinghai-Tibet Plateau regions experience relatively minimal human interference, where recent climatic trends of warming and increased humidity potentially foster favorable conditions for vegetation development (Zhang et al., 2023). Subsequent studies should emphasize a comprehensive approach that synthesizes regional climatic contexts, geomorphological characteristics, and anthropogenic impacts to elucidate the differential GPP response mechanisms across various geographical zones. Such investigations would offer valuable scientific insights and policy guidance for promoting sustainable ecosystem management practices.

## 5 Conclusion

To understand the complex response of GPP in various regions of China to compound hot extremes and drought, the relationship between compound hot extremes and drought and GPP was analyzed. This study first calculated the interannual and spatial variations of STI, SPEI and GPP from 2001 to 2020. Then, the correlations among STI, SPEI, the frequency of compound hot extremes and drought and GPP were analyzed. Finally, the influence intensity of STI, SPEI and compound hot extremes and drought (SPEI∩STI) on GPP was analyzed. The key findings are as follows:

(1) The average annual GPP in China gradually increased from Northwest to Southeast during 2001-2020. Overall, GPP showed an upward trend, with the Central and South region experiencing the highest growth rate at 9.88 gC·m^-^²a^-1^. Temporally, STI across China’s six major regions displayed a slight upward trend, while spatially, most areas showed a significant increase, accounting for 92.77% of the regions. The SPEI indicated that from a temporal perspective, the SPEI of the six major regions in China remained basically unchanged during 2001-2020; from a spatial perspective, the proportion of areas in China where the SPEI increased was 44.54%.

(2)The frequency of compound hot extremes and drought events in China’s six major region sranged from 5% to 23% during 2001-2020. Spatially, the frequency in most areas of each region was mainly moderate. The average frequency of the events was highest in North China (14.4%), followed by Northeast China (14.2%), Central and South China (13.7%), Southwest China (13.6%), East China (13.2%), and Northwest China (13.1%).

(3)This study analyzed the correlations between STI, SPEI, the frequency of compound hot extremes and drought events and GPP. The study revealed that STI was primarily negatively correlated with GPP, suggesting that rising temperatures adversely affect GPP, particularly in East China and Central South China. The study revealed that STI was primarily negatively correlated with GPP, suggesting that rising temperatures adversely affect GPP, particularly in East China and Central South China. The frequency of compound hot extremes and drought events was mainly negatively correlated with GPP, with stronger correlations observed in North China and Northwest China.

(4)Using the Geographical Detector, the study analyzed the relationships among various factors and obtained the q values for the impact of compound hot extremes and drought on GPP across the six regions: North China (0.51) > Central and South China (0.25) > East China (0.23) = Northwest China (0.23) > Northeast China (0.20) > Southwest China (0.16). Moreover, the q value for the effect of compound hot extremes and drought on GPP was higher than that of SPEI and STI individually, indicating a more severe impact on GPP when these conditions occur together.

## Data Availability

The raw data supporting the conclusions of this article will be made available by the authors, without undue reservation.
